# Co-expression network analysis of duplicate genes in maize (*Zea mays* L.) reveals no subgenome bias

**DOI:** 10.1186/s12864-016-3194-0

**Published:** 2016-11-04

**Authors:** Lin Li, Roman Briskine, Robert Schaefer, Patrick S. Schnable, Chad L. Myers, Lex E. Flagel, Nathan M. Springer, Gary J. Muehlbauer

**Affiliations:** 1 Department of Agronomy and Plant Genetics, University of Minnesota, Saint Paul, MN 55108 USA; 2 National Key Laboratory of Crop Genetic Improvement, College of Plant Science and Technology, Huazhong Agricultural University, Wuhan, 430070 China; 3 Department of Computer Science and Engineering, University of Minnesota, Minneapolis, MN 55455 USA; 4 Department of Agronomy, Iowa State University, Ames, IA 50011 USA; 5 Monsanto Company, Chesterfield, MO 63017 USA; 6 Department of Plant and Microbial Biology, University of Minnesota, Saint Paul, MN 55108 USA

**Keywords:** Gene duplication, Gene expression, Co-expression network, Regulatory divergence, Maize (*Zea mays* L.)

## Abstract

**Background:**

Gene duplication is prevalent in many species and can result in coding and regulatory divergence. Gene duplications can be classified as whole genome duplication (WGD), tandem and inserted (non-syntenic). In maize, WGD resulted in the subgenomes maize1 and maize2, of which maize1 is considered the dominant subgenome. However, the landscape of co-expression network divergence of duplicate genes in maize is still largely uncharacterized.

**Results:**

To address the consequence of gene duplication on co-expression network divergence, we developed a gene co-expression network from RNA-seq data derived from 64 different tissues/stages of the maize reference inbred-B73. WGD, tandem and inserted gene duplications exhibited distinct regulatory divergence. Inserted duplicate genes were more likely to be singletons in the co-expression networks, while WGD duplicate genes were likely to be co-expressed with other genes. Tandem duplicate genes were enriched in the co-expression pattern where co-expressed genes were nearly identical for the duplicates in the network. Older gene duplications exhibit more extensive co-expression variation than younger duplications. Overall, non-syntenic genes primarily from inserted duplications show more co-expression divergence. Also, such enlarged co-expression divergence is significantly related to duplication age. Moreover, subgenome dominance was not observed in the co-expression networks – maize1 and maize2 exhibit similar levels of intra subgenome correlations. Intriguingly, the level of inter subgenome co-expression was similar to the level of intra subgenome correlations, and genes from specific subgenomes were not likely to be the enriched in co-expression network modules and the hub genes were not predominantly from any specific subgenomes in maize.

**Conclusions:**

Our work provides a comprehensive analysis of maize co-expression network divergence for three different types of gene duplications and identifies potential relationships between duplication types, duplication ages and co-expression consequences.

**Electronic supplementary material:**

The online version of this article (doi:10.1186/s12864-016-3194-0) contains supplementary material, which is available to authorized users.

## Background

Gene duplication exists widely in nature, and can be divided into whole genome duplication, local (tandem) duplication, single gene transposition-duplication and chromosomal duplication [1]. Nearly all higher plants have experienced at least one whole genome duplication (WGD) and tandem/segmental duplications are also widely observed [2–4]. Gene duplication plays a vital role in evolution [5], and has been suggested to be more important than point mutations [6]. In vertebrates, large- and small-scale gene duplications contributed predominantly to the evolution and adaptive radiation of species [7]. Moreover, gene duplication followed by the diversity of genomic content and gene regulation is probably the major factor resulting in the speciation and adaptation in plants [8]. The additional copies of genes can introduce functional redundancy, which may promote evolutionary processes at either the coding or the regulatory level [9]. Duplicate copies may be affected by nonfunctionalization, where pseudogenization occurs, or neofunctionalization, where a novel gene function emerges, or subfunctionalization, where duplicate genes partition and share the ancestral gene function in different tissues and/or developmental stages [1, 6, 9–12].

Transcript abundance variation among duplicate genes is well-documented [13]. Gene duplication can increase gene expression diversity within and between species [14]. Duplicate genes can enable specialized expression differences in different tissues or developmental stages, as well as under different biotic or abiotic stress conditions [15–25]. Moreover, WGD was associated with co-expression regulatory network partitioning in *Saccharomyces cerevisiae*, resulting in more complex regulatory diversity [26]. In allohexaploid bread wheat, Pfeifer and colleagues explored the transcriptome dynamics and identified cell type- and stage-dependent genome dominance, indicative of genome interplay among different cell types in a polyploid cereal genome [27]. Thus, following duplication, there is a trend toward increased complexity and specialization among duplicate pairs and other interacting genes.

Maize provides a useful system to study the regulatory divergence of duplicate genes. Maize has undergone a recent WGD event ~5–12 Mya followed by whole genome rearrangement, which combined the duplicated chromosomes into a diploid genome containing 10 mosaic chromosomes [28]. The maize genome is divided into two distinct subgenomes, referred to as maize1 and maize2 [29]. Based on greater gene retention and higher expression level among homeologs, maize1 exhibits subgenome dominance over maize2 [29]. Widespread neofunctionalization was also observed between homeologs in maize1 and maize2 assuming that both ancient genomes were equal at the moment of WGD [30]. Purifying selection and dominant gene expression contributed to subgenome evolution after the recent WGD in maize [31]. In addition to the expression divergence permitted by WGD in maize, segmental gene duplication (i.e. tandem duplication and inserted duplication) was also shown to be related with the differential expression of maize genes with different tissue expression specificities [32–35]. However, the landscape of whole-genome regulatory divergence of maize WGD, inserted and tandem duplication events remains largely unexplored.

Co-expression network approaches provide insights into the patterns of transcriptome organization and suggest common biological functions for networked genes. Co-expression edges represent significant expression level correlations between genes based on expression profiles across a set of samples. Clusters of genes with co-expression edges are grouped into shared modules in the co-expression network. A number of studies have utilized diverse datasets (microarray data and RNA-seq data) to identify modules of genes with shared patterns of expression in plants [36–40]. There is also evidence that co-expression modules can be conserved across species [41, 36, 42]. In some cases, genes exhibiting coordinated expression across samples are biologically co-regulated [43]. Thus, co-expression modules have the potential to infer the regulatory network of genes. The availability of a maize genome sequence [44] and ample transcriptome datasets [45] provides an opportunity to explore the regulatory (co-expression) outcomes of duplicate genes. Of particular interest is the co-expression divergence of duplicated genes in maize 1 and maize 2 subgenomes. The functional divergence of duplicate genes, especially the subgenome interplay and divergence at the co-expression network level, was specifically addressed in this work.

Here, we developed a gene co-expression network from a transcriptome dataset consisting of 64 different tissues and stages from the reference inbred – B73. A total of 189 co-expression modules with at least ten genes were identified. We uncovered significant differentiation in co-expression networks among WGD, inserted and tandem duplications. Although the maize1 subgenome exhibits dominance in terms of gene retention and expression level, there is no significant bias of intra subgenome correlations within either of the two subgenomes. Our study provides a genome-wide classification of co-expression network divergence for different types of duplicate genes and uncovers similar levels of inter and intra subgenome interactions after WGD in maize.

## Results and discussion

### Development of a maize co-expression network

**Fig. 1:**
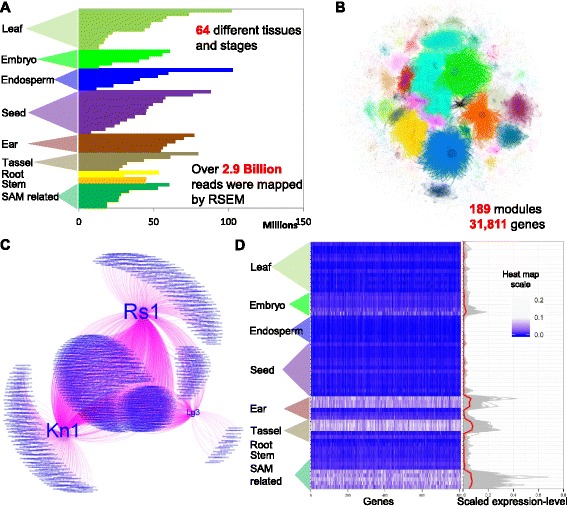
A co-expression network constructed in maize. **a** The distribution of uniquely-mapped reads in each tissue/stage. Detailed information on each tissue and developmental stage is available in Table 1. **b** A gene co-expression network for maize, including 189 modules with 31,811 genes. The co-expression network can be explored at the online database [46]. The blue module in the co-expression network shows a shoot apical meristerm specific sub-network. **c** A shoot apical meristem (SAM) module involving well-known SAM-expressed genes. Each node represents one gene, while each edge (line) linking two nodes indicates a significant co-expression correlation. *Kn1*, *Rs1* and *Lg3* represent the *Knotted1*, *Rough Sheath1* and *Liguleless3* genes, respectively. The size of gene labels is in accordance with the node size, which is further determined by the number of co-expressed genes. **d** The tissue-specific expression pattern of the SAM-specific module shown in C. Each row of the heatmap (*left panel*) indicates one tissue/stage and each column represents one gene. Each line of the plot (*right panel*) represents one gene; the red line shows the average scaled expression level across all tissues/stages

To explore the potential regulatory divergence of duplicate genes in maize, we utilized a RNA-Seq dataset (Fig. 1a and Table 1) from 64 different tissues and developmental stages of the reference inbred B73. These tissues and stages were classified into several distinct plant structures shown in Fig. 1a. More than 3.5 billion NGS reads were obtained and mapped to the maize B73 reference genome version 3 [44]. Over 2.9 billion reads were mapped to profile the transcriptome variation across different tissues and stages in maize (Table 1). Co-expression of genes was determined (see Methods for details) and several Z score thresholds were tested based on the biological means and statistical stringency (Additional file 1: Figure S1). Significant co-expression relationships (Z score >2.5) were adopted to construct a co-expression network, which contained 189 moderate-size modules of 31,811 genes (Fig. 1b). This network was integrated into the COB database [40] and can be explored by selecting the SAM dataset [46].

**Table 1:** Transcriptome Datasets used in our study

ID	Tissues/Stages	Tissue classification	# Total reads	% contaminating adaptor sequences	% bases >q20	Uniquely mapped reads in FGS gene space	% reads in FGS gene space	Reference
1	Trans	SAM related - embryo	22994564	5.24	95.87	17664753	76.82	[63]
2	LM	SAM related - LM	33567585	3.66	97.70	18748653	55.85	[63]
3	L1	SAM related - embryo	39027020	2.11	98.14	25877026	66.31	[63]
4	L14	SAM related - embryo	31676873	3.31	98.10	24636049	77.77	[63]
5	Col	SAM related - embryo	41066790	2.95	98.05	27532949	67.04	[63]
6	V5_Shoot_tip	SAM containing	37966015	5.49	96.71	31957110	84.17	[45]
7	V3_Stem and SAM	SAM containing	75700900	7.24	90.86	58077428	76.72	[45]
8	V1_4D_PE_Stem_SAM	SAM containing	68096357	5.66	95.80	58736028	86.25	[45]
9	V5_First_elonagetd_internode	stem	50620425	5.42	96.78	42021210	83.01	[45]
10	V9_Fourth_elongated_internode	stem	49883803	4.68	96.64	42811552	85.82	[45]
11	V1_4D_PE_Primary_root	root	34338211	5.38	96.03	28431679	82.80	[45]
12	6DAS_GH_Primary Root	root	75974682	7.89	94.81	58789326	77.38	[45]
13	tassel_stg1	tassel	41685877	1.38	99.38	37715842	90.48	[64]
14	tassel_stg3	tassel	44907570	1.60	99.08	39810029	88.65	[64]
15	tassel_stg2	tassel	33527176	1.28	98.97	29884294	89.13	[64]
16	R1_Anthers	tassel	50390993	6.51	95.22	42255501	83.86	[45]
17	V13_Immature_tassel	tassel	67777936	4.83	96.86	57328334	84.58	[45]
18	V18_Meiotic_tassel	tassel	90322080	4.17	96.99	76409461	84.60	[45]
19	V18_Immature_cob	ear	63065877	4.27	96.92	51946957	82.37	[45]
20	ear_tip	ear	19469355	3.85	97.91	17259171	88.65	[64]
21	ear_mid	ear	22832854	1.66	98.69	20181747	88.39	[64]
22	ear_base	ear	22257472	2.06	99.34	19599692	88.06	[64]
23	R1_Pre-pollination_cob	ear	78890387	3.61	97.56	65827854	83.44	[45]
24	R1_Silks	ear	85297444	3.47	97.73	73722387	86.43	[45]
25	16DAP_Whole_seed	seed	36090184	5.88	96.18	30262739	83.85	[45]
26	24H_Germinating Seed	seed	67115301	9.53	93.24	50590975	75.38	[45]
27	14DAP_Whole_seed	seed	57197138	8.00	94.15	49994899	87.41	[45]
28	10DAP_Whole_seed	seed	59980127	6.77	95.05	51983114	86.67	[45]
29	12DAP_Whole_seed	seed	55057984	7.51	94.26	47658798	86.56	[45]
30	18DAP_Pericarp	seed	45315693	8.06	94.50	37642629	83.07	[45]
31	2DAP_Whole_seed	seed	50626444	7.31	94.38	43151312	85.23	[45]
32	18DAP_Whole_Seed	seed	54296830	9.77	92.49	47308022	87.13	[45]
33	6DAP_Whole_seed	seed	53677071	10.33	92.65	45188276	84.19	[45]
34	8DAP_Whole_Seed	seed	58148634	10.14	92.10	48258631	82.99	[45]
35	20DAP_Whole_Seed	seed	76914729	7.66	94.53	60236045	78.32	[45]
36	22DAP_Whole_Seed	seed	71960325	8.36	93.25	61230630	85.09	[45]
37	24DAP_Whole_Seed	seed	92168996	7.53	94.03	80572513	87.42	[45]
38	4DAP_Whole_Seed	seed	100145919	8.22	93.97	84635434	84.51	[45]
39	14DAP_Endosperm	endosperm	47165528	5.74	95.93	41130552	87.20	[45]
40	16DAP_Endosperm	endosperm	43133667	24.17	81.93	36465697	84.54	[45]
41	24DAP_Endosperm	endosperm	44105309	6.98	95.06	39097597	88.65	[45]
42	22DAP_Endosperm	endosperm	50805946	8.39	93.89	44051966	86.71	[45]
43	20DAP_Endosperm	endosperm	73033574	8.58	93.76	56588796	77.48	[45]
44	18DAP_Endosperm	endosperm	71722169	9.68	92.92	62724952	87.46	[45]
45	12DAP_Endosperm	endosperm	105002559	3.78	97.71	90746296	86.42	[45]
46	Pro	embryo	29413276	4.14	96.73	12517310	42.56	[63]
47	16DAP_Embryo	embryo	42474548	25.27	81.01	35238795	82.96	[45]
48	20DAP_Embryo	embryo	46773334	8.18	93.69	39847251	85.19	[45]
49	22DAP_Embryo	embryo	54593430	8.38	94.22	46407023	85.00	[45]
50	18DAP_Embryo	embryo	66441077	8.93	93.22	55947651	84.21	[45]
51	24DAP_Embryo	embryo	69915678	7.27	94.61	59151359	84.60	[45]
52	VT_Thirteenth_Leaf	leaf	37375422	14.22	88.85	28643636	76.64	[45]
53	V9_Eleventh_Leaf	leaf	34439420	9.38	92.76	29562400	85.84	[45]
54	R2_Thirteenth_Leaf	leaf	44695525	11.87	91.31	35886544	80.29	[45]
55	V9_Thirteenth_Leaf	leaf	44205877	14.04	89.31	36100685	81.66	[45]
56	V9_Eighth_Leaf	leaf	50771168	13.85	89.38	42468259	83.65	[45]
57	V9_Immature_Leaves	leaf	43514876	25.26	81.13	35013880	80.46	[45]
58	V1_4D_PE_Pooled_Leaves	leaf	52523629	8.80	93.61	44510623	84.74	[45]
59	V3_Topmost_leaf	leaf	55571667	6.22	96.08	46448356	83.58	[45]
60	V7_Bottom_of_transition_leaf	leaf	53754587	5.08	96.09	46018705	85.61	[45]
61	V5_Bottom_of_transition_leaf	leaf	62309853	5.04	97.24	53863310	86.44	[45]
62	V5_Tip of stage-2 Leaf	leaf	79560349	6.88	94.70	63185974	79.42	[45]
63	V7_Tip_of_transition_leaf	leaf	84671628	5.19	95.87	71020587	83.88	[45]
64	6_DAS_GH_Coleoptile	leaf	121128249	6.43	94.92	103474057	85.43	[45]

Gene co-expression networks can be used to identify modules based on tissue/stage-specific expression patterns. An example is a shoot apical meristem- (SAM) related expression module (Fig. 1c), which contains several well-characterized genes *Rough sheath1* (*Rs1*) [47, 48], *Knotted1* (*Kn1*) [49, 50] and *Liguleless3* (*Lg3*) [51]. A total of 790 genes were co-expressed with *Kn1*, *Rs1* and *Lg3*. There are 254 genes that were co-expressed with all three genes, while 401 genes were only correlated with one of the three genes. This SAM-related specific expression module is composed of genes with a distinct expression pattern across 64 different tissues/stages (Fig. 1d). Many (55 %) of the modules with at least 10 genes exhibited significant GO enrichments (Additional file 2: Table S1). This gene co-expression network provides a resource to explore the regulatory divergence of duplicate genes in maize.

### Identification of duplicate genes in maize

**Fig. 2:**
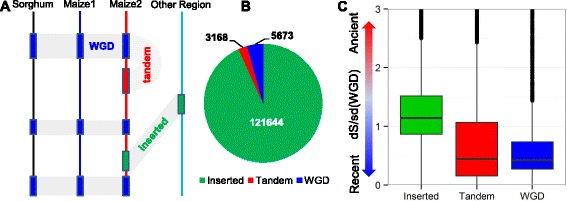
Identification of duplicate genes and their relationship with duplication age. **a** Schematic diagram of duplication types in the maize genome relative to sorghum. Duplications were classified into three major patterns: whole genome duplication (WGD), tandem duplication and inserted duplication (located in non-syntenic positions). Boxes represent genes and the grey area shows the homologous relationship of genes between maize subgenomes or between species. “Other region” refers to a non-syntenic genomic location. **b** The number of duplicate gene pairs in different duplication types. **c** The synonomous mutation (dS) distribution of duplicate genes across different duplication types. sd(WGD) shows the standard deviation of dS of WGD pairs

The Needleman-Wunsch algorithm with BLOSOM62 scoring matrix implemented in NCBI blast package [52] was used to identify paralogous duplicate gene pairs (See Methods) among the 39,323 annotated maize genes from the maize reference genome version 3 [44]. In total, 130,485 duplicate pairs were classified as whole genome duplications (WGD), local tandem duplications (tandem) or single gene insertions (inserted) based on grass pan genome synteny blocks [53] (Fig. 2a-b and Additional file 3: Table S2). Genes from inserted duplications are prevalent in maize, which may be due to the widespread transposon elements and transposition events in the maize genome [44]. The rate of synonymous mutations (dS) was used as a proxy for duplication age for each gene pair (Fig. 2c). Duplicate genes from WGD have the lowest mean dS and smallest variance, as expected with a single WGD event and subsequent divergence. Inserted duplicates exhibit a higher mean of dS, indicating inserted duplication occurred earlier than the recent WGD and tandem duplications (*P* < = 2.2e-16), while tandem duplicates show a higher variance, implying that they occurred continually over a long period of time (Fig. 2c).

### Co-expression network divergence of duplicate genes

Gene duplication, which generates functional redundancy, can allow duplicates to diverge in a coding sequence or expression-level manner [9]. These duplicate divergences could play an important role in species evolution and environmental adaptation [1, 5, 8, 12]. To dissect these divergences, we examined expression patterns of the duplicate genes in the co-expression network. Maize provides an ideal system to study the co-expression network divergence of duplicate genes because of the clear history of “WGD” [28, 29] and ample available transcriptome datasets [45].

**Fig. 3:**
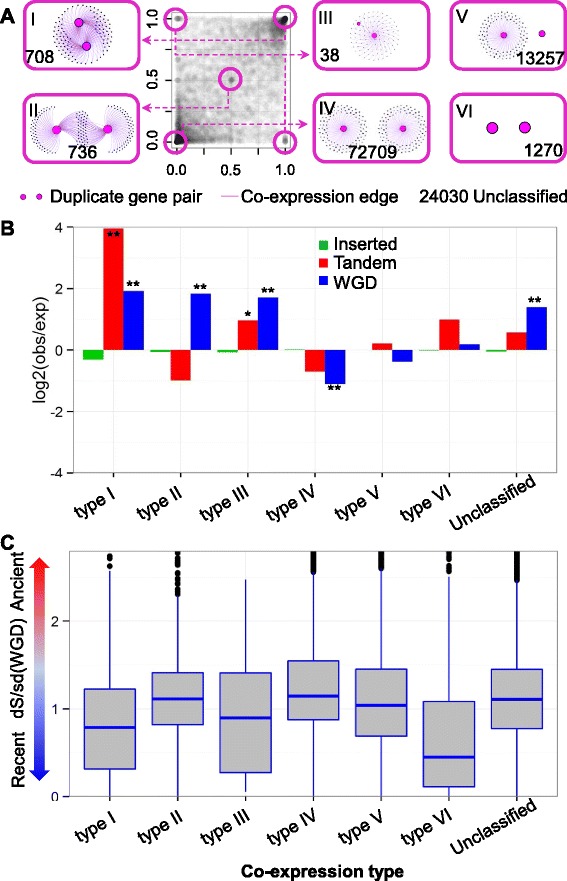
Regulatory divergence patterns of duplicate genes in co-expression network partitioning. **a** Classification of different types of co-expression patterns for duplicate genes. The proportion of shared genes (x axis and y axis) of a specific gene module is defined as the number of shared correlated genes between two duplicates divided by the total number of correlated genes for one of the duplicate genes. Each dot represents a pair of duplicate genes. A total of six co-expression regulatory patterns were classified and an example of the type of networks is visualized in the pink boxes along with the number of genes in each pattern. The other duplicate pairs were designated as “unclassified”. **b** The enrichment or depletion of genes in each co-expression pattern was assessed for each of the categories of gene duplications. The log_2_ ratio of observed to expected proportion of genes in each class is shown and “**” are used to indicate significant enrichment (P < 0.01). The expectation was calculated based on the proportion of duplicate pairs shown in Fig. 2b. **c** The co-expressed divergence patterns was related to the age (indicated by dS) of the duplications. The synonymous mutation rate (dS) divided by the standard deviation of dS among WGD pairs is shown for each class of co-expression relationships

The duplicate pairs were classified into several types based on the relative co-expression relationships of both genes (Fig. 3a). For each pair of duplicate genes (gene1 and gene2), two statistics were determined, the proportion of common neighbors in the gene1 co-expression module and the proportion of common neighbors in the gene2 co-expression module. These two proportions were then used to characterize each duplicate gene pair (Fig. 3a). Four patterns of relative co-expression relationships were classified: type I – completely overlapping edges; type II- partial overlap of edges; type III - minimal overlap of edges; and type IV – non-overlapping edges for the pair (Fig. 3a). In addition, two other types of pairs were classified: type V – one gene without edges; and type VI – no edges for both members in the pair (Fig. 3a). The remaining pairs of duplicates genes, which could not be classified into any of the above six co-expression patterns but with at least 10 edges for both duplicate genes, were designated as “unclassified”. About 11 % of the duplicate pairs had no neighbors for either one or both of the members of the pair (types V and VI). The majority (56 %) of the genes that had edges for both members of the pairs have little or no overlap of edges (type IV). Duplicate genes from all six distinct co-expression groups exhibit significant functional GO enrichment (Additional file 4: Table S3). The duplicate genes with type I co-expression show strong GO enrichments in sexual reproduction, response to oxidative stress and response to chemical stimulus, while type VI is enriched for transcriptional regulators (Additional file 4: Table S3). Interestingly, genes from type IV to VI, which show extreme co-expression difference between duplicates, have GO enrichment of responses to environmental changes. Taken together, our co-expression network allowed us to classify duplicate genes in maize, providing a resource to understand potential gene regulatory divergence after gene duplication.

The relationship between co-expression patterns and duplication types was investigated. The WGD gene pairs were most enriched in types I, II III, and the unclassified group, and are depleted in types IV and V, suggesting that many WGD pairs have common neighbors for both genes. The tandem pairs were enriched for type I genes with completely overlapping co-expression neighbors (Fig. 3b). Although duplicate pairs classified as inserted were not significantly enriched in any of the co-expression groups, they are slightly depleted in groups I, II, III, VI and unclassified, and slightly increased in group IV co-expression groups, suggesting that inserted pairs tend to have one or both members with no strong co-expression relationships or that these pairs lack common neighbors. The association between duplicate co-expression network divergence and duplication types is consistent with the results in rice and Arabidopsis [13, 18]. These results agree with the scenario where whole genome duplicates tend to maintain intact promoter regions and further express both copies, while duplicate genes from “inserted” duplication events are prone to lose promoter regions and reduce the correlated expression of duplicates [54]. Our result is also in agreement with the DNase I footprint variation between WGD and tandem duplicate genes in Arabidopsis, where whole genome duplicates have more footprints than do tandem duplicates and further allow whole genome duplicates to form more complex regulatory networks than tandem duplicates [55].

We also examined the relationship between co-expression patterns and duplication age, which was estimated using synonymous mutations (dS). The different patterns for co-expression relationships of the duplicate genes exhibit differences in duplication age (Fig. 3c). Types I, II and III (together) tend to have younger duplicates (*P* < 2.2E-16, compared to types IV and V). Type V has slightly older duplicates while type IV has the oldest duplicates. This may reflect that older duplications are more likely to have diverged in co-expression partners as type IV has the most divergent co-expression partners. Surprisingly, the youngest duplications seemed to be enriched for type VI. This may be due to duplicates from the youngest duplications lacking the time to set up the co-expression network with other functional genes. Overall, this result indicates that duplication age may play a role in the co-expression partitioning of duplicate genes. The longer duplicate pairs are retained, the more likely the breakdown and partitioning of their shared co-expression network.

Besides the exploration of co-expression divergence from a biological angle described above, we also employed metrics from graph theory to measure co-expression divergence in terms of the edge number in the shortest path between duplicates (a path with minimized weights of its constituent edges between two nodes), node clustering coefficient (a measure of how close its neighbors are to being a complete graph) and local node connectivity (the minimum number of edges needed to remove to eliminate all paths from one gene to its duplicate counterpart). Consistently, duplication types were related to co-expression divergences (Additional file 5: Figure S2A, B and C). WGD duplicates were more likely to be connected in the co-expression network, while duplicates from inserted duplication tended to be singletons. However, tandem duplicates showed more similarity to inserted pairs in terms of the edge number in the shortest path and local node connectivity. Moreover, younger duplications related to less co-expression divergence than older pairs (Additional file 5: Figure S2D and E).

### A substantial number of inter subgenome correlations were uncovered after whole genome duplication

**Fig. 4:**
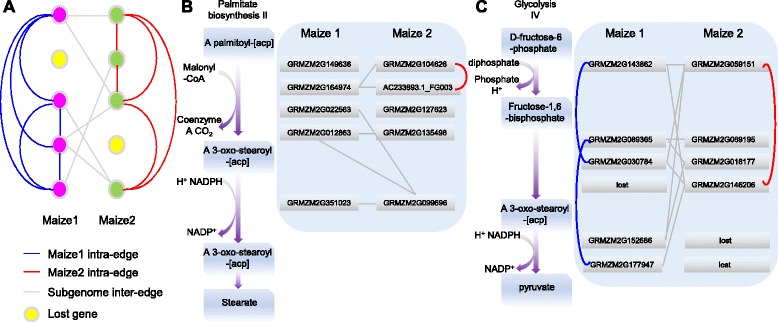
Co-expression network fractionation within metabolic pathways after maize whole genome duplication. **a** A schematic of a hypothetical co-expression network after a WGD event is illustrated to define potential relationships. Maize1 and maize2 intra-edges indicate the co-expression correlations among genes from the same subgenome. Subgenome inter-edges represent the co-expression relationship between genes which are from different subgenomes. The “lost” genes (in yellow) are those that have been fractionated after the recent WGD event and are missing from one of the subgenomes. Maize1 genes are shown in pink circles, while maize2 genes are in green circles. **b**-**c** Examples of co-expression networks in the stearate biosynthetic pathway where all genes were retained and the glycolysis IV pathway in which some duplicate pairs have been fractionated resulting in lost genes. Genes on the right panel encode enzymes corresponding to each pathway on the left

A pre-grass WGD shared among all grass genomes, the radiation of the grasses, and a maize lineage-specific recent WGD occurred during maize evolution [53]. Given the long period of divergence and substantial fractionation for the first two ancient large-scale genomic evolution events, we focused our analyses on the two maize subgenomes (maize1 and maize2) that were generated by the recent WGD event. Prior to a WGD event, genes from the ancestral genome would likely interact with each other to carry out their biological function, which could be inferred by co-expression correlation as clustering modules [43]. After a WGD event, co-expression correlations between genes from the same ancestral genome can be classified as subgenome intra edges and co-expression correlations between genes from the duplicated genomes can be classified as subgenome inter edges (Fig. 4a). Previous studies indicate that WGD can result in co-expression relationships among genes that are more likely to be restricted to pairs of genes from the same subgenome (i.e. intra edge correlations; Fig. 4a) [26]. Maize1 is the dominant subgenome, which ought to maintain more intra genome co-expression relationships, while the non-dominant maize2 loses functional relationships due to both gene loss and decreased gene expression. Our prediction is that maize1 will show more intra edge relationships than maize 2 intra edge or inter edge relationships between the two subgenomes. Thus, maize1 and maize2 provide a useful system to examine divergence of co-expression relationships after a WGD event and assess the extent of intra and inter edge correlations.

To characterize potential changes in the co-expression networks for metabolic pathways after a recent WGD event, we first assessed the co-expression network of 428 pathways annotated by maizeCyc [56]. The stearate biosynthetic pathway, which has retained all homeologs and the glycolysis IV pathway, which was partially fractionated following the WGD event were selected as examples for visualization (Fig. 4b-c). The extant maize stearate biosynthetic network contains seven co-expression edges and six of the seven co-expression edges are inter subgenome in nature, providing evidence of prevalence of inter subgenome correlations following the WGD event (Fig. 4b). The glycolysis IV pathway also has more inter than intra subgenome edges (Fig. 4c). Both examples demonstrate the occurrence of inter subgenome correlations after the recent WGD event.

**Fig. 5:**
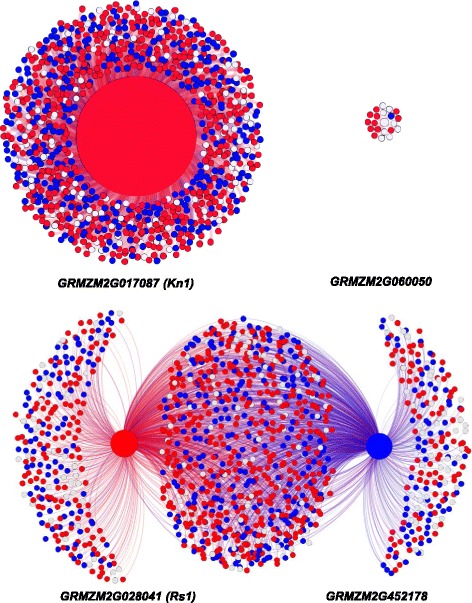
Co-expression divergence patterns for two duplicate pairs of well-known functional genes. (A) The *Kn1* duplicate pair of *GRMZM2G017087* (*Kn1*) and *GRMZM2G303381* showed the co-expression type IV pattern. (B) The *Rs1* duplicate pair of *GRMZM2G028041* (*Rs1*) and *GRMZM2G452178* showed co-expression type II variation. Red indicates the gene is from subgenome maize1, while blue indicates that the gene is from subgenome maize2. Grey means the gene could be anchored on either subgenome. Only genes anchored distinctly to maize1 or maize2 were used to quantify the level of inter- and intra- subgenome correlations

To further assess the relative prevalence of inter- and intra subgenome correlations in metabolic pathways, we explored the co-expression network divergence for 32 pathways, which have more than seven co-expression edges among genes from maize1 and maize2 subgenomes (Additional file 6: Table S4). No significant difference was observed in the density (fraction of co-expression relationships observed over the possible number of pairs) of maize1 intra-edges and maize2 intra-edges (*P* = 0.85, t-Test: Paired Two Sample for Means), suggesting limited divergence of co-expression relationships between the two subgenomes. Interestingly, there are equivalent number of inter subgenome edges to the total number of maize1 and maize2 intra-edges (*P* = 0.65 for absolute edge number; Additional file 6: Table S4). Furthermore, the overall proportion of inter and intra subgenome edges is not significantly different (*P* = 0.56 for the normalized edge number normalized by the number of possible pairs). We further dissected the co-expression network divergence for the duplicate pairs of the *Kn1* [49, 50] and *Rs1* [47, 48] genes. We observed co-expression network divergence for both duplicate pairs, the *Kn1* pair showed the gene co-expression type IV pattern, while *Rs1* exhibited the type II pattern (Fig. 5). However, the probability of intra and inter subgenome edges for both cases are not significantly different (*P* = 0.46 and 0.86 for *Kn1* and *Rs1* pair, respectively).

The prevalence of inter subgenome interactions was also assessed for all WGD pairs to determine whether the observations from metabolic pathways were representative of genome-wide trends. Separate analyses were performed for retained gene pairs and for genes that only retain the maize1 or maize2 gene. In both cases, the permutation analysis with the same number of genes and edges indicates no significant difference between the proportion of maize1 and maize2 intra-edges and a similar level of inter subgenome edges as intra subgenome edges (*P* = 0.83). Meanwhile, the contingency table analyses showed similar levels of inter subgenome and intra subgenome correlations (*P* = 0.72). Taken together, WGD in maize was accompanied by a large number of inter subgenome correlations, but in contrast exhibiting an equivalent level of intra subgenome correlations. These results are in contrast to the subgenome partitioning observed in yeast, whereby more intra subgenome correlations were observed [26]. This may be due to the fact that yeast is a single cell where all genes can directly respond to various environmental challenges. In more complex genomes, gene interactions were more likely to be regulated by transcription factors (hub genes), which may adjust the pathway in a more synergistic manner. If we assume most modules of ancestral genomes looked much like extant networks (i.e. same genes involved and same number of edges), loss of genes would remove intra-edges, and to complete metabolic pathways, these would be replaced by inter-edges. This may account for why there were large numbers of inter subgenome correlations observed given the widespread gene loss in maize subgenome2 [29]. Importantly, we do not know the ancestral state of the co-expression network prior to WGD event, so we could not postulate retention of ancestral correlations from newly evolved ones.

Ancient WGDs or paleopolyploids are widespread in flowering plants in the evolutionary history of different clades [2–4]. Comparisons of syntenic regions in *Arabidopsis thaliana*, *Zea mays*, and other flowering plants resulting from the most recent WGDs have uncovered the existence of biased gene content [29, 53, 54, 57–59]. This biased gene content between duplicate genomic regions could result from either more gene gain or more gene loss in a specific duplicate or one of the parental subgenomes [1]. One proposed model to explain the biased gene content is that the expression dominance of duplicate genes in one subgenome could make the less expressed copies in the other subgenome selectively neutral [29]. Based on the dominance of the maize1 subgenome, which would retain more biological function while maize2 would lose biological function due to gene loss and decreased expression level, we expected that maize1 subgenome would exhibit more functional dominance than maize2 in terms of more maize subgenome 1 intra edges. Unexpectedly, our co-expression network analyses identified similar levels of intra edge genome correlations in maize1 and maize2. In addition, we observed that there was a similarity in the frequency of intra and inter edge correlations, indicating that gene expression in the maize subgenomes was integrated very quickly even after the recent WGD. This result is robust to choice of cutoff for the detection of connecting genes in the co-expression network.

### No subgenome asymmetry was observed in maize transcriptional network

We constructed a *de novo* transcriptional network including 189 modules (subnetworks) involving 31,811 annotated maize genes. Of these co-expression modules, there were 48 modules with more than 20 classified maize1 and maize2 genes, however, only two modules showed maize1 subgenome dominance where significantly more maize1 genes were enriched (Adjusted *P* value < = 0.05). The proportion (4 %) of subgenome dominant modules is significantly lower than that (92 %) in allohexaploid bread wheat [27] (Additional file 7: Table S5). Hub genes, which are connected with thousands of other genes in network, may play a critical role in biological function of organisms. Furthermore, 1000 permutation tests of node degree in the maize co-expression network uncovered 525 highly connected genes in the network (hub genes) (Additional file 8: Table S6). However, these hub genes were not significantly more likely to show a subgenome bias (Chi-Square Test; *P* = 0.13), which is also different from that in wheat. Unlike wheat, the co-expression network in maize exhibited no subgenome asymmetry, which may be due to the relative older age of the maize whole genome duplication [44, 60]. Maize tetraploidy occurred between 5 and 12 million years ago, while allohexaploid genome bread wheat has experienced much less time (2.5 ~ 4 million years) to diverge [61]. Compared to wheat, the increased time maize had to merge subgenomes resulted in a genome that is highly integrated from a transcriptional viewpoint. Taken together, our results indicate that the maize genome does not have subgenome dominance in terms of transcriptional networks. However, our study provides a comprehensive landscape of co-expression divergence of duplicate genes after WGD in maize.

## Methods

### Transcriptome dataset in maize

All transcriptome datasets were publicly available and downloaded from NCBI Sequence Read Archive [45, 62–64]. A total of 64 experiments generated by next generation sequencing (NGS) were obtained from different tissues or development stages of maize reference inbred B73 (Table 1). The transcriptome dataset consists of both single-end reads and pair-end reads with read length ranging from 50 to 110 bp. Each experiment (tissue/stage) had 2 to 3 biological replicates. Each biological replicate was analyzed separately and the average normalized expression level of all the biological replicates was obtained to represent the expression-level of specific tissue/stage.

### Transcriptome profiling and co-expression network construction

After downloading all the transcriptome datasets, trimmomatic [65] was employed to remove all the adapter sequences. Then, low quality sequences were removed using Fastx-Toolkit [66]. High quality NGS reads of each biological replicate for each tissue/stage were mapped onto annotated gene region space of the maize reference genome (AGP v3) [44] using RSEM [67] with parameters *“-p 8 --bowtie2 --estimate-rspd --append-names --output-genome-bam* ”. The statistic “TPM” (Transcripts Per Million) was adopted as the proxy of normalized expression-level. A matrix (39,323 annotated maize genes X 64 transcriptome datasets) of a transcriptome profiling dataset across 64 different tissues/stages was used for further analyses.

We considered a gene was expressed if it had TPM > 0 in at least three tissues or TPM > 5 in at least one tissue. A total of 37,649 genes were determined to be expressed across 64 different tissues/stages of maize reference inbred B73. To reduce the weight of highly expressed genes on correlation coefficients, we transformed TPM values using inverse hyperbolic sine function, which compressed large values while preserving the relative magnitude of small values [45]. Co-expression networks were constructed by calculating Pearson correlation coefficients between all pairs of genes (37,649 × 37,649):

$$ {\boldsymbol{R}}_{ij}=\boldsymbol{P}\boldsymbol{C}\boldsymbol{C}\left({E}_i,\ {E}_j\right) $$

Where *i*, *j* = 1, …, 37,649 and *i* ≠ *j*. The set of correlations was then transformed by Fisher transformation [68], which yields approximately normal distribution [38]:

$$ \boldsymbol{Z}=\frac{1}{2}\boldsymbol{ln}\frac{1+\boldsymbol{R}}{1-\boldsymbol{R}} $$

Fisher transformed values were then standard normalized such that the resulting co-expression edge distribution had a mean of zero and a standard deviation of one. A set of cutoffs of Z score (1.5, 2.0, 2.5 and 3.0) was used as the threshold for the detection of significant edge (interaction) between genes. The co-expression networks were created and analyzed using the Sleipnir C++ library [69]. The software Cytoscape 3.0.2 [70] was used for visualization of the co-expression networks. The co-expression network could be explored through the COB database [46]. Due to the memory limitation of local computers, only a small fraction of co-expression connections that users query can be rendered. However, the user can download the full co-expressed gene list using “Table View” of the COB database.

### Duplicate genes and the identification of expression-level variation and co-expression divergence pattern

Maize paralogous duplicate genes were identified using NCBI blast + [52], which adopted the Needleman-Wunsch algorithm with the BLOSOM62 scoring matrix. The candidate paralogous pairs were extracted using the cutoff *E* value < =1.0E-05 based on the protein sequence alignment of all maize annotated genes. Furthermore, if the proportion of aligned protein amino acid length to the full protein length is more than 40 % for both genes, the significant gene pair was considered as a paralogous duplicate. Then, the duplicate genes were compared and merged with syntenic gene blocks in maize [53]. For the paralogous duplicates, the protein sequence was translated into aligned codons, and further codeml of the PAML software package [71] was used for the calculation of synonymous mutation rate (dS) with its default parameter sets. Only duplicate pairs, of which both genes were expressed across 64 different tissues/stages, were kept for further analyses of co-expression network divergence.

Co-expression network divergence was examined by comparing the sharing neighbors between two duplicates. First, by exploring the co-expression networks, we summarized the number of nodes (correlated genes) of duplicate genes. Then, we computed the proportion of common neighbors (same correlated genes) for the members from any pair of duplication. The proportion of shared correlated genes of a specific gene module is defined as the number of shared correlated genes between two duplicates divided by the total number of correlated genes for one of the duplicate genes. Specifically, for a paralogous duplicate pair (gene1 and gene2), the statistic gene1common represents the proportion of common neighbors with gene2 for gene1, while gene2common indicates the proportion of common neighbors with gene1 for gene2. Both gene1common and gene2common range from 0 (without any common neighbors) to 1 (sharing all the neighbors) in the co-expression network. For the paralogous pairs where both duplicates have at least 10 neighbors (correlated genes), if $\sqrt{{\left(1- gene1 common\right)}^2+{\left(1- gene2 common\right)}^2}\le 0.1$, type I – completely sharing neighbors with each other was classified; if $\sqrt{{\left(0.5- gene1 common\right)}^2+{\left(0.5- gene2 common\right)}^2}\le 0.1$, type II – partial sharing of neighbors was classified; if $\sqrt{{\left(1- gene1 common\right)}^2+{\left(0- gene2 common\right)}^2}\le 0.1$, or $\sqrt{{\left(0- gene1 common\right)}^2+{\left(1- gene2 common\right)}^2}\le 0.1$, type III – minimal sharing of neighbors was classified; if $\sqrt{{\left(0- gene1 common\right)}^2+{\left(0- gene2 common\right)}^2}\le 0.1$, type IV – non-sharing neighbor for the pair was classified; otherwise, the paralogous pairs where both duplicates have at least ten neighbors (correlated genes) were designated as “unclassified”. If one duplicate has at least ten neighbors while the counterpart has no neighbors, such paralogous pairs were classified as type V. If both duplicates have no neighbors (singleton), such paralogous pairs were classified as type VI. A total of seven co-expression regulatory patterns (type I ~ VI and unclassified), were identified, while other paralogous duplicate pairs which do not satisfy the above criteria were excluded in our further analyses. Gene Ontology enrichment analyses for the genes from different co-expression divergence groups were performed using AgriGO [72].

Furthermore, to detect the co-expression divergence using graph theory, we also adopted the shortest network path, edge connectivity of a duplicate pair, and local clustering coefficient of genes in the co-expression network for the representation of co-expression divergence in the further analyses. The calculation was conducted using igraph R package [73].

We employed a set of Z score cutoffs (1.5, 2.0, 2.5 and 3.0) for the classification of co-expression divergence. Given the peak of proportion of type IV at around 2.0, the statistic significance, and the relative less number of unclassified co-expression type (Additional file 1: Figure S1), we employed Z score cutoff of 2.5 for the construction of maize co-expression network and further analyses.

### Duplication type, age and their relationships with regulatory divergence of duplicate genes

Duplication type and the manner that duplication occurred were obtained by analyzing maize updated syntenic gene blocks [53]. Three major duplication types were identified: “WGD” is defined as whole genome duplication; “tandem” for tandem duplication; and “inserted” for duplicate genes located in non-syntenic genomic regions (Fig. 2a). Synonymous mutation rate, which is an indicator of duplication age, was calculated using PAML [71]. All the relationship analyses were conducted in R [74].

### Co-expression edge fractionation in maize transcriptional network

Maize metabolic pathways were downloaded from MaizeCyc 2.2 [56, 75]. The metabolic pathways with at least two pairs of duplicate genes generated by recent WGD event were kept for the co-expression fractionation analysis. Maize1 intra edges, maize2 intra edges and inter subgenome edges were counted and summarized to identify different types of edges in each metabolic pathway. Paired two sample t-test was conducted on 32 metabolic pathways with at least seven edges for the comparison between maize1 intra edges, maize2 intra edges and inter subgenome edges. The overall genome-wide trend of co-expression edge fractionation was investigated based on two different sets of WGD duplicate genes: one is the retained gene pair, and the other is genes that have been subjected to fractionation. In both cases the frequency of inter subgenome correlations and the frequency of intra subgenome interactions were calculated and compared with each other. Permutation analysis with the same number of genes and edges was performed to test if the observation that more inter subgenome edges than intra edges is significant. Similar analyses with less stringent Z score cutoffs (1.5 and 2.0) were conducted and showed consistent results.

### Subgenome enrichment test in maize co-expression network modules

The mcl markov cluster algorithm was employed to distinguish co-expression network modules with default parameters [76]. According to maize pan genome information [53], we summarized the number of maize1 genes and maize2 genes, and then applied a Chi-square test to discriminate if specific subgenome genes were enriched in any co-expression network modules with at least 20 maize1 and maize2 genes. The significance of the Chi-square test was adjusted through Bonferroni correction.

Hub genes, which had significantly more connected genes than the average in the network, were analyzed in our study. The degree (the number of the co-expressed genes) of each gene in the maize co-expression network was obtained by in-house perl scripts. Further, 1000 permutation tests with the same number of genes (nodes) and significant correlations (edges) were conducted to obtain the cutoff (α = 0.05) of highly connected genes (hub genes) in the maize co-expression network. A cutoff of 2706 was obtained for the identification of 525 hub genes in our study. Finally, Chi-square tests on the 525 hub genes were performed to check if these genes were enriched in maize1 or maize2. These tests were conducted after taking into account the gene number differences between maize1 (15,231) and maize2 (9553).

## Conclusions

We developed a co-expression network for the maize inbred line B73 from 64 different tissues/stages B73 and used the network to explore the expression fate of duplicate genes. There are four key findings from our work: (1) WGD, tandem and inserted gene duplications exhibit different regulatory divergence; (2) co-expression variation was greater in older duplicate genes than younger duplicates; (3) more co-expression divergence was observed in inserted duplications and and this divergence was also related to the age of the duplication; and (4) maize1 and maize2 exhibit similar levels of intra and inter subgenome correlations, indicating that there is no subgenome dominance in the network.

## Additional files

Additional file 1: Figure S1. The proportion of different co-expression divergence patterns identified across different cutoffs of Z score. (PDF 64 kb)
Additional file 2: Table S1. GO enrichment for genes from different co-expression modules. (XLSX 365 kb)
Additional file 3: Table S2. Duplicate genes. (XLSX 19372 kb)
Additional file 4: Table S3. GO Enrichment for duplicate genes with different co-expression types. (XLSX 21 kb)
Additional file 5: Figure S2. Co-expression divergence in terms of shortest network path, edge connectivity of a duplicate pair, and local clustering coefficient. (A) WGD duplicates had significantly shorter path edges than inserted and tandem duplicate genes. (B) WGD duplicates had significantly higher edge connectivity than inserted and tandem duplicate genes. (C) Maize1 and maize2 genes had significantly lower local clustering coefficient than non-syntenic genes (usually generated by inserted and tandem duplications). (D) and (E) Shortest network path and edge connectivity of a duplicate pair were associated with synonymous mutation rate (dS). (PDF 740 kb)
Additional file 6: Table S4. Co-expression divergence in metabolic pathways. ^a^-the number of duplicate pairs in the metabolic pathway; ^b^-the number of co-expression edges in the metabolic pathway; ^c^-the possibility of maize1 intra-edges; ^d^-the possibility of inter-subgenome co-expression edges; ^e^-the possibility of maize2 intra-edges; ^f^-the possibility of total intra-edges in metabolic pathway. (XLSX 13 kb)
Additional file 7: Table S5. Subgenome gene enrichment in co-expression network modules. (XLSX 12 kb)
Additional file 8: Table S6. Subgenome gene enrichment in the set of hub genes. (XLSX 522 kb)

## Abbreviations

DB: DataBase
Mya: Million year ago
WGD: Whole genome duplication
