# Supplementary figures and images for: Co-expression network analysis of duplicate genes in maize (Zea mays L.) reveals no subgenome bias
Source: BMC Genomics. 2016 Nov 4;17:875. doi: 10.1186/s12864-016-3194-0 (PMC5097351; doi:10.1186/s12864-016-3194-0)

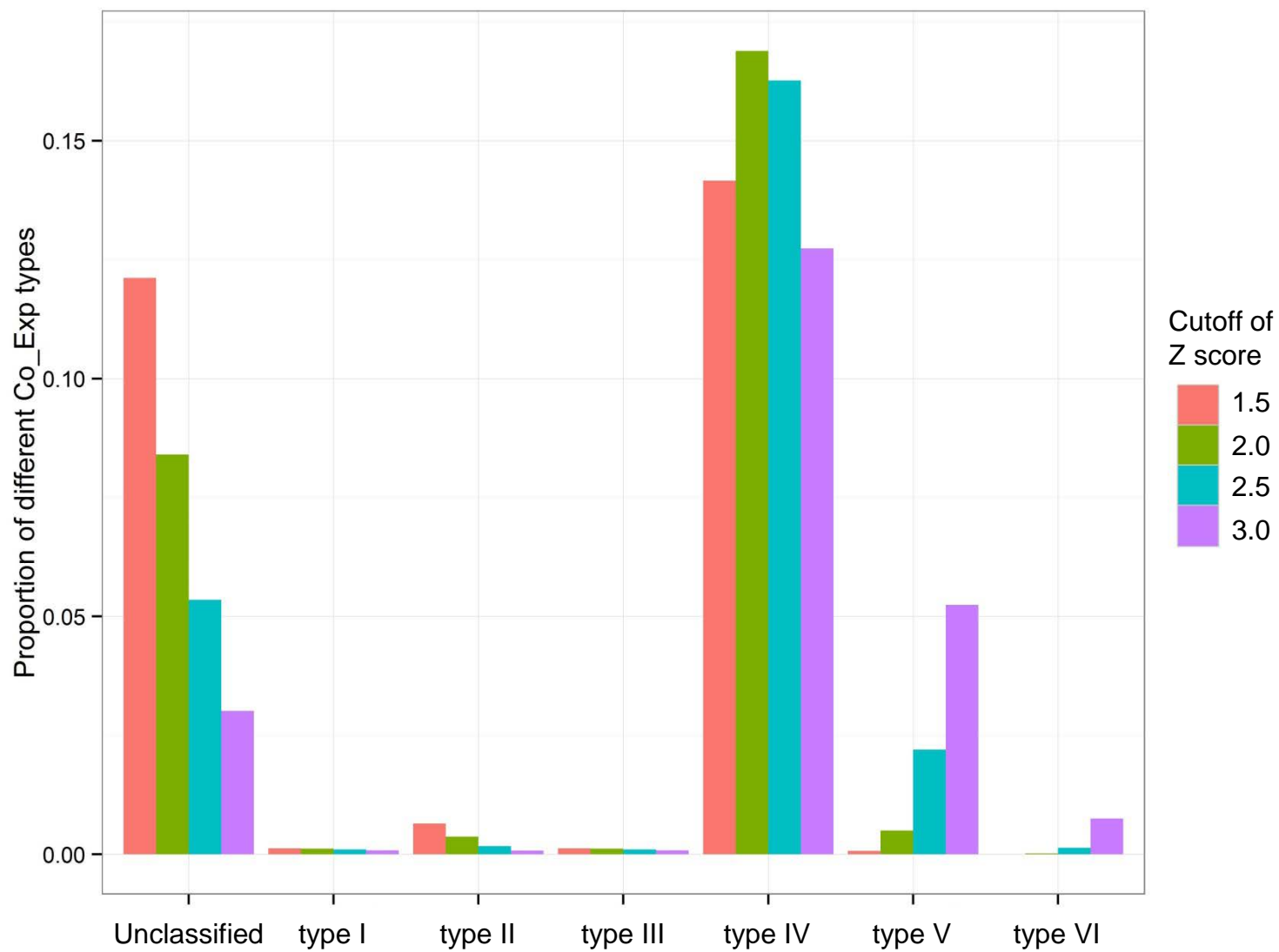

Supplement: Additional file 1: Figure S1. — The proportion of different co-expression divergence patterns identified across different cutoffs of Z score. (PDF 64 kb) [file 12864_2016_3194_MOESM1_ESM.pdf]

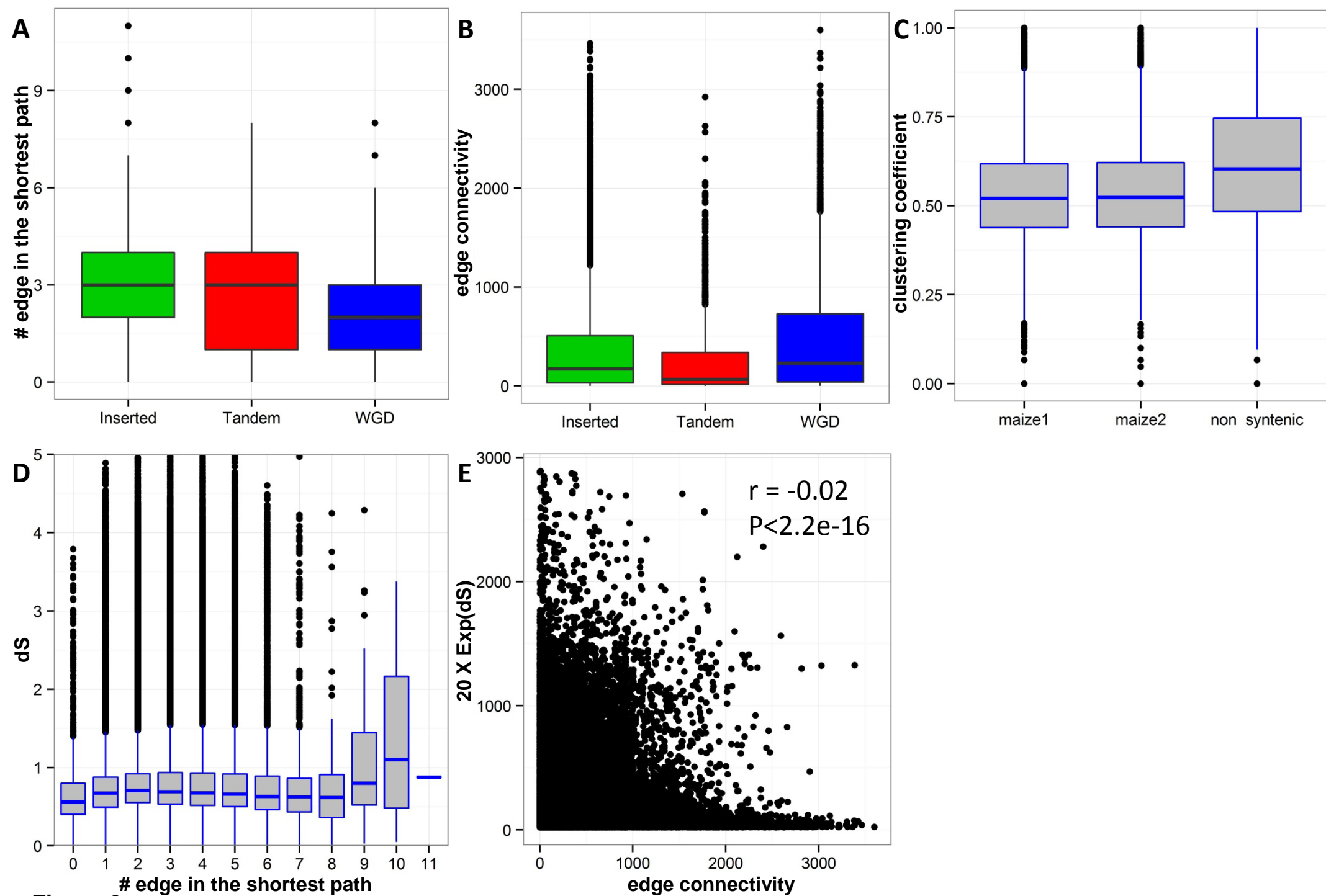

**Figure 6**

Supplement: Additional file 5: Figure S2. — Co-expression divergence in terms of shortest network path, edge connectivity of a duplicate pair, and local clustering coefficient. (A) WGD duplicates had significantly shorter path edges than inserted and tandem duplicate genes. (B) WGD duplicates had significantly higher edge connectivity than inserted and tandem duplicate genes. (C) Maize1 and maize2 genes had significantly lower local clustering coefficient than non-syntenic genes (usually generated by inserted and tandem duplications). (D) and (E) Shortest network path and edge connectivity of a duplicate pair were associated with synonymous mutation rate (dS). (PDF 740 kb) [file 12864_2016_3194_MOESM5_ESM.pdf]
